# Factors associated with implementation of a multicomponent responsible beverage service program – results from two surveys in 290 Swedish municipalities

**DOI:** 10.1186/1747-597X-8-11

**Published:** 2013-03-04

**Authors:** Björn Trolldal, Ulrika Haggård, Karin Guldbrandsson

**Affiliations:** 1 Department of Clinical Neuroscience, STAD, Stockholm County Council/Karolinska Institutet, Box 6031, SE-102 31, Stockholm, Sweden; 2 Department of Medical Epidemiology and Biostatistics, Karolinska Institutet, Stockholm, Sweden; 3 Department of Public Health Sciences, Karolinska Institutet, Stockholm, Sweden; 4 Centre for Violence Prevention/Swedish National Correction Service, Box 23000, SE-104 35, Stockholm, Sweden; 5 Swedish National Institute of Public Health, SE-831 40, Östersund, Sweden

**Keywords:** Implementation, Responsible beverage service program, Community-based interventions, On-licensed premises, Violence

## Abstract

**Background:**

The purpose of this study was to investigate which factors affected the implementation of a multicomponent Responsible Beverage Service (RBS) program in 290 Swedish municipalities and whether the amount of such factors influenced the level of implementation of the program.

**Methods:**

This study used variation in the presence of implementation-promoting factors to predict the level of implementation of the RBS program in municipalities throughout Sweden. The presence of such factors and the level of implementation of the program were studied by means of two surveys in all Swedish municipalities (N=290). Logistic regression and Spearman’s correlation analyses were used to analyze the relationship between implementation-promoting factors and the level of implementation of the RBS program.

**Results:**

The response rates of the two surveys were 96% and 98%, respectively. One main finding was that program fidelity was low. Only 13% of the municipalities surveyed had implemented the RBS program as a whole, as stated in the specification of requirements. In municipalities reporting a higher amount of implementation-promoting factors, a significantly higher level of implementation of the program was shown. Evaluation and feedback was the only factor that correlated significantly with the level of implementation of the RBS program as a whole.

**Conclusion:**

Evaluation and feedback constitutes an important implementation-promoting factor also in complex programs like the RBS program. Program fidelity is significant for the outcome of an intervention and must be a major focus of the implementation processes.

## Background

### Introduction

Alcohol consumption constitutes a main risk factor for health and contributes substantially to the global burden of disease [1]. Previous research has revealed a strong relationship between alcohol consumption and violence [2]. A crucial factor in alcohol-related violence is the level of intoxication [2-4]. Moreover, associations between public violence and drinking, especially spirits and beer, in on-licensed premises [5-7], as well as between the number of licensed premises in a community and acts of public violence have been documented [8,9]. Several studies in Sweden have shown that 75%–80% of the perpetrators of violent crimes were under the influence of alcohol at the time the crimes were committed [6], and the attributable fraction has been estimated at around 50% [2,5,6]. In recent decades, both the number of on-licensed premises and the duration of their opening hours have increased markedly in Sweden.

Population-based strategies that focus mainly on general affordability and availability and interventions directed toward consumers already at risk have proven to be cost-effective means of reducing alcohol-related harm [10,11]. Interventions that target the drinking context as well as the supply of alcohol can be effective means to reduce the risk of alcohol-related harm [12,13]. To implement such interventions, multicomponent activities driven by national and local governments are needed [14]. One such intervention is the multicomponent RBS program [15].

However, very few multicomponent RBS programs that aim to reduce violence associated with consumption of alcohol at on-licensed premises have been implemented and evaluated. One example, apart from the program implemented in Stockholm described below, is the Safety Action Projects in Queensland, Australia, where significant effects were found on observed aggression [12,16]. Single-component server-training programs have been implemented with mixed effects on servers’ attitudes and serving practices [17], but few of these programs aimed to reduce violence. One exception is the Safer Bar project in Ontario, Canada, where a significant effect was found on aggression [18]. The effects on violence of enhanced police enforcement and supervision of on-licensed premises have been mixed [12,19,20]. However, in a review regarding dram shop liabilities, strong evidence was found for effects on motor vehicle crash deaths, particularly on alcohol-related crash deaths [21].

### The multicomponent responsible beverage service program in Sweden

The main purpose of the Swedish multicomponent RBS program is to reduce violence associated with the consumption of alcohol at licensed premises by means of reducing the level of customer intoxication. The program was set up as a research project with an experimental area and a control area in the central part of Stockholm, the capital of Sweden. An evaluation of the RBS program showed a significant decrease in the number of police-recorded violent crimes, primarily assaults committed between 10:00 p.m. and 6:00 a.m., with 29% in the intervention area as compared to the control area [15]. The study period was 1998–2001. Very strong effects were found on the serving of alcohol to pseudo-intoxicated patrons as well [22-24]. A cost-effectiveness analysis, based on the results of the evaluation of 1998-2001, showed a cost-saving ratio of 1:39 [11].

After the initial evaluation, the RBS program grew in popularity, and the number of municipalities using the program increased during 2003 and 2004. In 2004, the Swedish National Institute of Public Health was commissioned by the government to spread the RBS program to municipalities throughout the country. Two years later, Sweden’s 21 county administrative boards were appointed to assist in implementing the program. One project coordinator was employed at each county administrative board to oversee implementation. Dissemination of the RBS program culminated in 2006 and 2007 when 84 and 60 municipalities, respectively, adopted the program. In 2008, representatives from 263 of Sweden’s municipalities (90%) stated that they had implemented the RBS program, in full or in part [25].

A new evaluation was conducted in 2010 after dissemination of the program throughout Sweden; again, a significant effect was found on police recorded assaults [25]. For each of the three main program components (described below) used in the municipalities, the effect was 3%, on average. Consequently, if all three main components were used, the effect was approximately 9%.

The RBS program consists of several components, of which this study focuses on the three main ones: education of restaurant personnel (RBS training), the presence of a community-coalition steering group, and the supervision of on-licensed premises [25]. The RBS training, which normally takes two days, comprises instruction on the medical effects of alcohol, the Alcohol Act, alcohol-related violence, drug problems at on-licensed premises, and conflict management, followed by an exam. The aim of the community-coalition steering group is to create a platform for communication between the owners of on-licensed premises and the authorities so that alcohol-related problems at licensed premises can be discussed and resolved. Finally, the supervision visits at on-licensed premises are designed to ensure compliance with provisions regarding the serving of alcoholic beverages, particularly to intoxicated patrons and minors. According to the program, it is important that the supervision visits takes place mainly late at night, when the serving of alcohol is most intense. It is also important that municipal employees and the police conduct supervision visits in cooperation as well as that police conduct supervision visits on their own [26].

The Swedish Alcohol Act specifies that municipal licensing boards are responsible for the handling of licensing matters. Both the municipality and the police department are responsible for the supervision of licensed premises, while the municipality alone is responsible for sanctions regarding infractions committed at these premises. The purpose of the act is to ensure that sales of alcohol are conducted in such a manner as to prevent harmful effects in a broad sense as much as possible. The focus is on overserving and sales to minors.

Although research on implementation strategies has increased during the past decades, the evidence to support practice is insufficient [27,28]. Factors affecting the process of implementation, in this study labelled *implementation-promoting factors*, however, have been suggested in the literature [29-33].

### Purpose of the study

The purpose of this study was to analyze the implementation of a complex, multicomponent public health program—the RBS program—in Swedish municipalities. Two specific questions were addressed:

1. Are there any correlations between the presence of implementation-promoting factors and the level of implementation of the RBS program?

2. Is there a dose-response effect, i.e., does a higher amount of implementation-promoting factors result in a higher level of implementation of the RBS program?

## Methods

### Surveys

The analyses in this study are based on data from two surveys conducted in all Swedish municipalities (N=290). The first, conducted at the turn of the year 2008-2009, studied to what degree municipalities had implemented the RBS program [25]. The second survey was conducted a year later, at the turn of 2009-2010. The purpose of this survey was to ascertain if implementation-promoting factors had been present in the municipalities at the time the RBS program was implemented. In both surveys, respondents were civil servants working with alcohol-related issues in the municipalities. In order to maximize response rates the project coordinators at the county administrative boards were asked to distribute the surveys and give several specific reminders for each municipality.

The study was approved by the Regional Ethical Review Board in Stockholm (EPN) (protocol no. 2008/1041-31/5 and 2010/736-32/5).

### Inclusion criteria

Two criteria for municipalities to be included in the analysis part of this study were formulated: First, the municipality must have initiated the RBS program no later than 2008. Second, the municipality must contain on-licensed premises open during evenings. With the assistance of Sweden’s 21 county administrative boards, every municipality in the country was examined regarding fulfillment of these criteria. Of 290 municipalities, 235 qualified for inclusion.

### The first survey—level of implementation of the RBS program

Although no manual has been developed to describe the details of the RBS program, a brochure has been published in which the main features of the program are described [26]. These features were interpreted and used to create a set of requirements for the municipalities to fulfill in order to be approved for each of the three main components of the program (RBS training, a community-coalition steering group, and supervision). Questions in the surveys were asked to document the extent to which these requirements were fulfilled. The requirements were as follows:

#### RBS training

1. RBS training should have been conducted for employees at on-licensed premises in the municipality.

2. The duration of the training should have been at least two days.

3. The training should have included the following five instructional components: the medical effects of alcohol consumption, the Alcohol Act, alcohol-related violence, drug problems at licensed premises, and the handling of conflict at the premises, followed by an exam.

4. At least one training session with employees from licensed premises in the municipality should have taken place during the past two years.

5. The number of on-licensed premises that sent employees to training sessions had to amount to at least 30% of the total number of on-licensed premises in the municipality.

#### Community-coalition steering group

1. A community-coalition steering group for the concrete work conducted in accordance with the RBS program should have been present in the municipality or in a group of municipalities.

2. The municipality, the police department, and owners of on-licensed premises should have been represented in the group.

3. The community-coalition steering group should have conducted at least two meetings during the past year.

#### Supervision

To ascertain whether or not supervision in a given municipality was in line with the main features of the program, supervision was divided into three categories: supervision visits that were carried out (a) after 11:00 p.m., (b) by the municipality and the police department in collaboration, and (c) solely by the police department.

The supervision component of the RBS program had to satisfy three requirements:

1. At least two of the three categories of supervision described above had to be conducted during a year.

2. The number of on-licensed premises visited during one year, for each category, had to correspond to at least 20% of the number of licensees in the municipality.

3. The number of supervision visits at on-licensed premises had to have either increased during the course of the program or remained on the same level as it was when the municipality started to work in accordance with the RBS program.

#### Index measuring implementation of the RBS program as a whole

Municipalities that had satisfied the requirements for implementation obtained 1 point for each program component. To measure the degree to which each municipality had implemented the RBS program as a whole, the number of points was summarized in the form of an index, with each municipality receiving from 0 to 3 points.

### The second survey—implementation-promoting factors

The second survey was used to ascertain the presence of certain implementation-promoting factors at the time of implementation of the RBS program in the municipalities. Factors related to perceived local needs [29], characteristics of the intervention [32,33], the implementation process [30,32], decisions made [29], and participants involved in the process [31] were scrutinized (see Table 1). Several potential implementation-promoting factors, for example, the extent to which the program conformed to the values of the implementing organization [32,33] and factors related to the organizational context within which the program was to be implemented [29] were considered inappropriate to capture by surveys and were not included in this study.

**Table 1:** Implementation-promoting factors, survey questions, answer alternatives, mean values, and standard deviations

**Implementation-promoting factors, variables, and survey questions**	**Answer alternatives, maximum number of points, mean values, and standard deviations**
1. Needs [29]	
*Local needs:*	
Did any of the problems below exist in your municipality before Responsible Beverage Service or a similar program was implemented?	Yes=1
	No=0
• Service to minors	Maximum number of points: 4
• Service to noticeably intoxicated patrons	Mean: 1.6
• Violence and injuries related to beverage service in on-licensed premises	Standard deviation: 1.2
Has any particular event occurred in your municipality, county, or the country as a whole which has caused you to want to develop your work to diminish beverage service to minors or noticeably intoxicated patrons, or to diminish violence and injuries related to beverage service in on-licensed premises?	
2. Characteristics of the intervention [32,33]	
*Experienced program qualities:*	Yes=2
In your opinion, is Responsible Beverage Service or any similar program	To some extent=1
• An effective program to diminish violence and injuries related to beverage service in on-licensed premises?	
• An effective program to diminish beverage service	
• To minors?	No=0
• An effective program to diminish beverage service to noticeably intoxicated patrons?	(The same alternatives were given for each question under the factor characteristics of the intervention.)
• A cost-effective program?	
• A program that is easy to use?	
• A program where the effects are easy to observe?	
• A program which is easy to adapt to local circumstances?	
	Maximum number of points: 14
	Mean: 11.1
	Standard deviation: 2.5
3. The implementation process [30,32]	
*Support:*	
Did those who worked with Responsible Beverage Service or a similar program receive any of the following kinds of support? (Multiple choices are possible.)	Oral information=1
	Written information=1
	Web-based information=1
	Education on the program=1
	Coaching=1
	Optional advice=1
	Feedback=1
	Other=1
	No support was offered=0
	Maximum number of points: 8
	Mean: 3.7
	Standard deviation: 1.9
*Early involvement of practitioners:*	
Did any of the persons who later worked with the program in practice participate early in the process which led to implementing it? (Multiple choices are possible.)	Alcohol administrators at the municipality=1
	Supervision personnel=1
	The police=1
	Owners of on-licensed premises=1
	Other=1
	No=0
	Maximum number of points: 5
	Mean: 2.0
	Standard deviation: 1.2
*Evaluation and feedback*	
Questions to those municipalities where studies have been conducted to follow up work with the program:	One or more intoxication studies=1
	One or more studies of minors=1
1. What kinds of studies have been done in your municipality? (Multiple choices are possible.)	One or more studies of statistics of violence=1
	One or more rounds of interviews with owners of on-licensed premises=1
2. How were the results of the follow-up presented? (Multiple choices are possible.)	In one or more memoranda=1
	In one or more reports=1
	In one or more press releases=1
	At one or more spoken presentations for politicians=1
	At one or more spoken presentations for civil servants/practitioners=1
	Other=1
	The results have not been presented=0
	Maximum number of points: 10
	Mean: 1.9
	Standard deviation: 2.6
4. Decisions [29]	
*Political decision about the program:*	
A question to those municipalities where a decision has been made to use the Responsible Beverage Service or a program with the same or a similar content:	*The question referred to a political decision, and the alternatives were*
	Municipal executive board=1
At what level was the decision made?	Local council=1
	Local council committee=1
	Civil servant=0
	Maximum number of points: 1
	Mean: 0.6
	Standard deviation: 0.5
*Mentioned in guidelines:*	
Is the work in accordance with Responsible Beverage Service or a similar program mentioned in the following documents in your municipality? (Multiple choices are possible.)	Supervision plan=1
	Alcohol and drug policy program=1
	Instructions for licensing=1
	Public health plan=1
	Other documents of policy or plans for action=1
	No=0
	Maximum number of points: 5
	Mean: 1.1
	Standard deviation: 1.1
5. Opinion leaders [31]	
*Opinion leaders*:	
Has there been a single person or a group of persons in your municipality that has been the driving force behind implementing Responsible Beverage Service or a similar program? (Multiple choices are possible.)	Yes, one or more administration managers=1
	Yes, one or more police=1
	Yes, one or more politicians=1
	Yes, owners and staff of on-licensed premises=1
	Yes, alcohol administrators at the municipality=1
	Yes, alcohol and drug prevention coordinators=1
	Yes, public health coordinators or the like=1
	Yes, other persons=1
	No=0
	Maximum number of points: 8
	Mean: 1.6
	Standard deviation: 1.1

#### Index measuring the total presence of implementation-promoting factors

Eight variables (local needs, experienced program qualities, support, early involvement of practitioners, evaluation and feedback, political decisions, guidelines, and opinion leaders) were used to capture the presence of five implementation-promoting factors (needs, characteristics of the intervention, the implementation process, decisions, and opinion leaders) in the municipalities (see Table 1).

In one of the analyses in the study, where the potential dose-response effect is analyzed, the implementation-promoting factors were summarized in an index giving a picture of the total presence of such factors in the municipalities. Each factor is represented by at least one variable. In order to avoid putting more weight on factors that have more than one variable, each factor was given a maximum value of ten points. Since there are five separate factors, the maximum number of points on this index is 50.

### Background variables

To control for other characteristics of the municipalities that might have affected implementation of the program, three relevant background variables relating to the size of the municipality, the potential amount of resources, and the density of on-licensed premises in the municipalities were included in the analyses. These were, respectively, the number of inhabitants 15 years of age and older, the mean income level, and the number of on-licensed premises per 10,000 inhabitants.

### Statistical methods

Logistic regression analysis was used in order to study the relation between the eight variables measuring the presence of implementation-promoting factors and the level of implementation of the RBS program. The dependent variables, i.e. the level of implementation of each main component of the RBS program as well as the program as a whole, were given two values in four separate analyses. Municipalities that had fulfilled all requirements for the implementation of a program component were allotted one point for that component; municipalities that had not were allotted zero points. Regarding implementation of the program as a whole, municipalities that had implemented all components were given one point, while the rest were given zero points.

The odds ratio specifies the degree to which the odds of implementation increased if the independent variable increased by one unit. However, because each independent variable has a different number of units, the odds ratios cannot be compared. Wald statistics were used to estimate whether an independent variable had a significant effect on the level of implementation (estimates not shown). Nagelkerke’s R^2^ was used as the coefficient of determination to measure the extent to which all variables in a regression model explained the variation in the level of implementation of the program among municipalities.

Spearman’s correlation coefficient was used in the univariate analysis of the correlation between the indices measuring the level of implementation-promoting factors and the level of implementation of the RBS program.

## Results

The response rates of the two surveys were high, 96% and 98%, respectively. Of the 235 municipalities that satisfied the inclusion criteria for this study, only 13.2% had implemented all three main components of the RBS program, and 17.0% had implemented none of these components (see Table 2). The mean value of the number of components implemented was 1.45 (of a maximum of 3 points).

**Table 2:** The level of implementation of the RBS program and the presence of implementation-promoting factors

**Level of implementation of the RBS program (0–3 points)**	**Number of municipalities (n=235)**	**Presence of implementation-promoting factors (0–50 points) (Std. dev.)**
0	40 (17.0%)	25.2 (5.1)
1	79 (33.6%)	26.7 (4.8)
2	85 (36.2%)	27.6 (5.8)
3	31 (13.2%)	29.1 (5.9)
	235 (100.0%)	

The most commonly implemented program component was the RBS training, implemented by 64.3% of the municipalities. The steering group and the supervision components were implemented by 33.6% and 47.7% of the municipalities, respectively (see Table 3).

**Table 3:** The level of implementation of each RBS program component in the municipalities

**Program component**	**Number of municipalities**
RBS training	151 (64.3%)
Steering group	79 (33.6%)
Supervision	112 (47.7%)

The number of implementation-promoting factors present was larger the larger the number of program components implemented in the municipalities. The mean value on the index was 29.1 (of a maximum of 50 points) among municipalities that had implemented all three components, compared to 25.2 among those that had not implemented any components of the RBS program (see Table 2).

The total amount of implementation-promoting factors was significantly correlated with the level of implementation of the RBS program as a whole, as well as with two of the three program components: community-coalition steering group and supervision (see Table 4).

**Table 4:** The correlation between the total number of implementation-promoting factors and the level of implementation of the RBS program as a whole as well as the three main components of the program

	**Correlation coefficient (r**_**s**_**)**
The program as a whole	0.22**
RBS training	0.05
Steering group	0.16*
Supervision	0.19**

(Spearman’s correlation coefficient) (n=235).

*p<0.05 **p<0.01.

The factor *evaluation and feedback* (an implementation process factor) had a significant effect on the level of implementation of the RBS program. This was apparent in analyses of the full program as well as in separate analyses of two of the three main components, namely RBS training and supervision (see Table 5). Of the 235 municipalities that fulfilled inclusion criteria, 95 both conducted follow-up studies and reported the results. The most common forms of feedback were oral presentation to politicians (55 municipalities), written reports (50 municipalities), and media reports (48 municipalities).

**Table 5:** Implementation-promoting factors related to the implementation of the RBS program, odds ratios

	**The program as a whole**	**RBS training**	**Steering group**	**Super-vision**
*Needs:*				
Local needs	0.84	0.93	0.96	1.02
*Characteristics of the intervention:*				
Experienced program qualities	1.11	0.99	1.09	0.98
*The implementation process:*				
Support	0.81	0.88	0.95	0.96
Early involvement of practitioners	1.22	1.11	1.42*	1.24
Evaluation and feedback	1.31**	1.20**	1.01	1.14*
*Decisions:*				
Political decision about the program	1.91	0.67	1.62	0.80
Mentioned in guidelines	1.10	0.85	0.88	1.21
*Opinion leaders:*				
Opinion leaders	0.92	0.95	0.88	1.13
*Background variables:*				
Level of income	0.99	1.00	0.99	1.00
Number of inhabitants	1.00	1.00	1.00*	1.00
Number of licensed premises/10,000 inh.	1.00	0.99	1.02	1.01
Constant	0.18	4.47	0.92	0.27
Nagelkerke R^2^	0.21	0.16	0.17	0.15

(n=235).

*p<0.05 **p<0.01. Test: Wald chi-square (df=1).

The factor *early involvement of practitioners* (another implementation process factor) was significantly associated with the level of implementation of the *community-coalition steering group* component, but not with implementation of the RBS program as a whole (see Table 5).

## Discussion

The response rate of the two surveys in this study was very high (98% and 96%, respectively). This was probably due to repeated municipality-specific reminders that were distributed by the project coordinator at each county administrative board.

Of the 235 municipalities that reported having worked fully or partly in accordance with the RBS program, 17.0% had implemented none of the program components. Furthermore, only 13.2% had implemented the program as a whole, as stated in the specification of requirements. Thus, program fidelity was low. One reason for this may be that the information provided was unsatisfactory when implementation took place, since only a simple brochure describing the RBS program was available.

Evaluation and feedback showed a significant correlation with implementation of the RBS program as a whole, as well as with the two main program components RBS training and supervision. This result is in accordance with a Cochrane review based on 140 studies from the health care sector, where Ivers et al. conclude that “audit and feedback generally leads to small but potentially important improvements in professional practice” [34].

Early involvement of practitioners was significantly associated with the presence of a community-coalition steering group, but not with the other two program components (RBS training and supervision of on-licensed premises). This finding is not surprising since involvement in a steering group often implies involvement in the planning phase of an implementation process.

A dose-response effect was shown in this study in that higher amounts of implementation-promoting factors in a municipality were correlated with higher levels of implementation of the RBS program. In a study of over 500 health-promoting and disease-preventing programs, Durlak and DuPre showed that higher levels of implementation-promoting factors lead to better implementation results [29]. On the other hand, Grimshaw et al. found no relationship between the number of implementation-promoting factors and effects of multifaceted interventions [28].

The findings of this study should be considered in the light of some limitations. First, it is possible that errors in recall or social desirability biases crept into the surveys of key informants, meaning that levels of implementation of the RBS program could be even lower than those estimated in our study. Second, some implementation-promoting factors were not included in this study because of the difficulty of capturing those factors through surveys. For example, one factor left out of the survey, after much consideration, involved the extent to which the program conformed to the values of the implementing organization [32,33]. Another area of questioning not included in this study related to the organizational context within which the program was to be implemented [29]. In order to measure such factors, a different and more extensive study design would be needed, including methods to measure organizational readiness. Finally, the survey measuring the presence of implementation-promoting factors was distributed one year after the survey that measured the level of implementation of the RBS program. Although respondents were asked to describe the presence of implementation-promoting factors during the time of implementation of the RBS program there is a risk of recall bias.

One final question is whether it is worth the effort to implement such a complex, multicomponent program as this RBS program? The answer is, in all probability, yes. As mentioned in the introduction, two evaluations in Sweden have shown significant effects on police-recorded violence, and the cost-effectiveness ratio based on the results of the first evaluation was large.

## Conclusions

The most important conclusions of this study are these:

•Program fidelity was weak. All the 235 municipalities in this study stated that they had implemented the RBS program, but only 13% applied all three main components of the program.

•Evaluation and feedback seems to be an important implementation factor in multicomponent and complex interventions like the RBS program.

•There seems to be a dose-response effect as regards implementation-promoting factors. That is, the more implementation-promoting factors there are, the higher the level of implementation will be, even when it comes to a highly complex program such as the RBS program.

## Competing interests

The authors declare that they have no competing interests.

## Authors’ contribution

The study design was developed by all the authors. BT and UH carried out the surveys, and BT performed the statistical analysis. All the authors contributed to drafting the manuscript. All the authors have read and approved the final manuscript.
